# Isolation Method and Characterization of Outer Membranes Vesicles of *Helicobacter pylori* Grown in a Chemically Defined Medium

**DOI:** 10.3389/fmicb.2021.654193

**Published:** 2021-06-02

**Authors:** Joana Melo, Vanessa Pinto, Tânia Fernandes, Ana R. Malheiro, Hugo Osório, Ceu Figueiredo, Marina Leite

**Affiliations:** ^1^i3S – Instituto de Investigação e Inovação em Saúde, Universidade do Porto, Porto, Portugal; ^2^Ipatimup – Instituto de Patologia e Imunologia Molecular da Universidade do Porto, Porto, Portugal; ^3^ICBAS – Instituto de Ciências Biomédicas Abel Salazar, Universidade do Porto, Porto, Portugal; ^4^Departamento de Patologia, Faculdade de Medicina da Universidade do Porto, Porto, Portugal

**Keywords:** *Helicobacter pylori*, bacterial outer membrane vesicles, defined growth medium, OMVs isolation method, proteomics

## Abstract

Outer membrane vesicles (OMVs) are small vesicles constitutively shed by all Gram-negative bacterium, which have been proposed to play a role in *Helicobacter pylori* persistence and pathogenesis. The methods currently available for the isolation of *H. pylori* OMVs are diverse and time-consuming, raising the need for a protocol standardization, which was the main aim of this study. Here, we showed that the chemically defined F12 medium, supplemented with cholesterol, nutritionally supports bacterial growth and maintains *H. pylori* viability for at least 72 h. Additionally, we developed an abridged protocol for isolation of OMVs from these bacterial cultures, which comprises a low-speed centrifugation, supernatant filtration through a 0.45 μm pore, and two ultracentrifugations for OMVs’ recovery and washing. Using this approach, a good yield of highly pure *bona fide* OMVs was recovered from cultures of different *H. pylori* strains and in different periods of bacterial growth, as assessed by nanoparticle tracking analysis, transmission electron microscopy (TEM), and proteomic analyses, confirming the reliability of the protocol. Analysis of the proteome of OMVs isolated from *H. pylori* F12-cholesterol cultures at different time points of bacterial growth revealed differentially expressed proteins, including the vacuolating cytotoxin VacA. In conclusion, this work proposes a time- and cost-efficient protocol for the isolation of *H. pylori* OMVs from a chemically defined culture medium that is suitable for implementation in research and in the biopharmaceutical field.

## Introduction

*Helicobacter pylori* infects about half of the human population and remains the most common chronic infection in the world (Hooi et al., 2017). This Gram-negative bacterium is acquired in childhood, and unless treated, it establishes a life-long infection of the host gastric mucosa. Persistent *H. pylori* infection is associated with various clinical outcomes, such as peptic ulcer disease and gastric cancer, both adenocarcinoma and mucosa-associated lymphoid tissue (MALT) lymphoma (Atherton, 2006; Cover and Blaser, 2009).

*H. pylori*-mediated diseases, as well as bacterial colonization, survival, and persistence in the gastric mucosa depend upon the concerted action of multiple virulence factors and mechanisms (Blaser and Atherton, 2004). The secretion of outer membrane vesicles (OMVs) emerged as an additional means to facilitate such processes in *H. pylori* infection. It has been shown that *H. pylori* OMVs are internalized and deliver their cargo into epithelial cells, resulting in cellular alterations, such as vacuolization, elongation, modulation of proliferation, and a pro-inflammatory immune response (Chatterjee and Das, 1967; Devoe and Gilchrist, 1973; Hoekstra et al., 1976; Fiocca et al., 1999; Heczko et al., 2000; Ismail et al., 2003; Chitcholtan et al., 2008; Parker et al., 2010). The full contribution of OMVs to bacteria-host and bacteria-bacteria interactions is yet to be disclosed, considering that their effects may extend to locations distant from the locally circumscribed bacterial niche (Kulp and Kuehn, 2010).

Outer membrane vesicles are a type of membrane vesicles (MVs) secreted by virtually all Gram-negative bacteria. They have a spherical structure ranging from 20 to 500 nm in size, formed through the blebbing of the bacterial outer membrane (OM), enclosing periplasmic and cytoplasmic proteins, genetic material, and lipids (Kulp and Kuehn, 2010). Although there are other types of MVs secreted by different organisms, OMVs are the only type of MVs secreted by *H. pylori* described so far (Deatherage and Cookson, 2012). Even though OMVs are naturally secreted, vesiculation is strongly influenced by the bacterial growth stage and various environmental and stress conditions (Katsui et al., 1982; Thompson et al., 1985; Willen et al., 2000; Olofsson et al., 2010). *H. pylori* vesiculation, in particular, was shown to increase as bacterial growth progresses from the logarithmic to the stationary phase, and to associate with the morphological transition into the coccoid shape (Willen et al., 2000; Olofsson et al., 2010).

The current methods described for the isolation of *H. pylori*-derived OMVs are diverse and time-consuming. They include several ultracentrifugation and/or density gradient centrifugation steps to purify and recover OMVs, after an initial low-speed centrifugation and culture medium filtration intended to remove the bacteria (Kim et al., 2014; Klimentova and Stulik, 2015). Besides being laborious, the existing methods rely on complex and chemically undefined media for bacterial growth, such as Brucella Broth (BB) or Brain Heart Infusion (BHI), owing to the widely held notion that *H. pylori* is a fastidious organism and requires such enriched media to grow (Keenan et al., 2000; Mullaney et al., 2009; Kaparakis et al., 2010; Zavan et al., 2019). Nonetheless, Testerman et al. have demonstrated that *H. pylori* has few nutritional requirements and can grow in the chemically defined Ham’s-F12 (F12) liquid medium. Furthermore, they have shown that supplementation of F12 with cholesterol, bovine serum albumin, or fetal bovine serum, can further enhance *H. pylori* growth (Testerman et al., 2001, 2006).

Here, we developed a simpler and faster method to isolate OMVs from *H. pylori*, based on a chemically defined medium for bacterial growth. This enables the standardization of the isolation method, which is pivotal for downstream applications. Accordingly, we used the F12 liquid medium supplemented with cholesterol for bacterial growth, and carried out the isolation of OMVs with one low-speed centrifugation and a 0.45 μm-filtration to remove bacteria while allowing the recovery of all size-range OMVs, one ultracentrifugation step for vesicle purification, and a last ultracentrifugation as a final wash. Following this approach, a high yield of pure and *bona fide* spherical *H. pylori* OMVs were recovered.

## Materials and Methods

### *Helicobacter pylori* Strains and Growth Conditions in TSA Plates

*Helicobacter pylori* strains 26695 (ATCC^®^ 700392, *cagA*^+^, *vacA* s1/m1), 60190 (ATCC^®^ 49503, *cagA*^+^, *vacA* s1/m1) and Tx30a (ATCC^®^ 51932; *cagA*^–^, *vacA* s2/m2) were routinely cultured in Trypticase^TM^ Soy Agar (TSA) supplemented with 5% Sheep Blood (Becton, Dickinson and Company, Franklin Lakes, NJ, United States) and incubated in a sealed jar with a microaerophilic atmosphere (GENBox microaer; bioMérieux S.A., Marcy l’Etoile, France) at 37°C for 48 h. Bacteria were sub-cultured for a maximum of 12 passages.

### *Helicobacter pylori* Growth in Liquid Cultures

*Helicobacter pylori* previously grown in TSA plates for 48 h was collected with 1 mL of either sterile Ham’s F12 with L-glutamine medium (#L0135-500; Biowest, Nuaillé, France) supplemented with 1× cholesterol (Gibco^®^, Thermo Fisher Scientific, Waltham, MA, United States) (hereafter designated as F12-cholesterol), or BBL^TM^ Brucella broth (BB; #211088; BD Biosciences, San Jose, CA, United States) supplemented with 5% fetal bovine serum (FBS; HyClone^TM^, GE Healthcare Life Sciences, United States) (abbreviated as BB-FBS). The optical density at 600 nm (OD_600_) was measured using a spectrophotometer (Genesys20; Thermo Fisher Scientific), in polystyrene cuvettes (Thermo Fisher Scientific). The culture medium of the corresponding bacterial culture was used as the blank solution to calibrate the spectrophotometer to 100% absorbance.

The initial OD_600_ of the bacterial suspension was adjusted to 0.02 (∼6 × 10^6^ colony forming units – CFUs/mL) in 200 mL of F12-cholesterol or BB-FBS. Bacteria were grown in a 500 mL Schott flask, placed in a sealed jar, under microaerophilic conditions, at 37°C, and with a constant rotation of 100 rpm (SI600 Large Shaking Incubator; Stuart, Staffordshire, United Kingdom) for the defined periods of time, 24, 48, 64, or 72 h. Bacterial growth was monitored at the referred time points by measuring the OD_600_ of the liquid bacterial cultures.

### Bacterial Viability

The viability of *H. pylori* grown in liquid media was assessed by two methods: CFUs counting and flow cytometry using the LIVE/DEAD BacLight Bacterial Viability Kit (Thermo Fisher Scientific), according to the manufacturers’ instructions.

For the CFUs counting, at the referred time points, 100 μL of bacterial suspension was collected, serially diluted by 10-fold in BB, and 10 μL of each dilution was plated on TSA plates, in quadruplicates. Plates were incubated in a sealed jar with a microaerophilic atmosphere, at 37°C for 48 h. At this time, colonies were counted and the number of CFUs/mL was calculated (CFUs/mL = number of colonies ÷ inoculum × dilution factor × 1,000 μL).

For the LIVE/DEAD assay, a bacterial suspension of 2 × 10^6^ cells was filtered through a 10 μm CellTrics^®^ filter (Sysmex Partec, Göerlitz, Germany) to remove aggregates. Bacteria were then stained with 0.5 nM SYTO9, a cell-permeant green fluorescent nucleic acid stain that labels both live and dead cells, and with 2 μg/mL propidium iodide, a nucleic acid red-fluorescent dye that labels non-viable cells with damaged membranes, for 15 min at room temperature (RT) and in the dark. Data acquisition was immediately performed on a FACSCanto II cytometer (BD Biosciences), and analyzed using the FlowJo^TM^ version 10 software (BD Biosciences). SYTO9^+^ stained bacterial cells were defined as SYTO9^+^PI^–^ (live) or SYTO9^+^PI^+^ (dead) and results are shown as the frequency of gated cells. SYTO9 and PI single stainings and unstained samples were used as controls.

### Isolation of OMVs From Bacterial Liquid Cultures

Isolation of *H. pylori*-derived OMVs was performed from the supernatant of the liquid bacterial cultures. At the specified time points of bacterial growth, 200 mL of bacterial cultures were subjected to low-speed centrifugation at 15,000 × *g*, for 15 min at 4°C, in a JA25-50 rotor (Avanti J-25; Beckman Coulter, Fullerton, CA, United States), to pellet bacteria. The bacteria-free supernatant was filtered through a 0.45 μm cellulose acetate bottle-top filter unit (Corning, NY, United States), and then OMVs were pelleted by ultracentrifugation at 200,000 × *g*, for 90 min at 4°C, in a 70Ti rotor (Optima XE-100; Beckman Coulter). OMVs from the different centrifuge tubes were pooled and washed once in 20 mL sterile 0.9% NaCl solution (saline) (Braun; Kronberg im Taunus, Germany) (200,000 × *g*, 90 min, 4°C), resuspended in 100 μL saline and frozen at −80°C until use.

### Transmission Electron Microscopy

The morphology and purity of *H. pylori* cultures grown in F12-cholesterol and of *H. pylori-*derived OMVs were confirmed by transmission electron microscopy (TEM). The presence of protein aggregates in the liquid media was also evaluated by TEM. For negative staining, 7 μL of sample (bacterial suspensions, OMVs or liquid medium) was mounted in Formvar/carbon film-coated mesh nickel grids (Electron Microscopy Sciences, Hatfield, PA, United States), and after the excess liquid was removed, 2 μL of aqueous 1% uranyl acetated solution (#22400; Electron Microscopy Sciences) were added onto the grids. For the ultrastructure analysis, OMVs samples were fixed in a solution of 2% glutaraldehyde (#16316; Electron Microscopy sciences) with 2.5% formaldehyde (#15713; Electron Microscopy sciences) in 0.1 M sodium cacodylate buffer (pH 7.4) for 1 h, at RT, and post fixed in 1% osmium tetroxide (#19190; Electron Microscopy Sciences) diluted in 0.1 M sodium cacodylate buffer. After ultracentrifugation (200,000 × *g*, 90 min, 4°C), the pellet was resuspended in Histogel^TM^ (HG-4000-012, Thermo Fisher Scientific) and then stained with aqueous 1% uranyl acetate solution overnight, dehydrated and embedded in Embed-812 resin (#14120; Electron Microscopy sciences). Ultra-thin sections (50 nm thickness) were cut on a RMC Ultramicrotome (PowerTome, United States) using Diatome diamond knifes (DDK, Wilmington, DE, United States), mounted on mesh nickel grids (Electron Microscopy Sciences), and stained with uranyl acetate substitute (#11000; Electron Microscopy Sciences) and lead citrate (#11300; Electron Microscopy Sciences) for 5 min each. Negative staining samples and thin sections were examined under a JEOL JEM 1400 transmission electron microscope (JEOL, Tokyo, Japan) and images were digitally recorded using a CCD digital camera Orius 1100W (Tokyo, Japan).

### Scanning Electron Microscopy

For scanning electron microscopy (SEM) analysis, 5 mL of bacterial suspension from F12-cholesterol cultures were pre-fixed in 2.5% glutaraldehyde solution, diluted in 0.2 M sodium phosphate buffer (0.2 M Na_2_HPO_4_⋅2*H*_2_O and 0.2 M NaH_2_PO_4_⋅*H*_2_O in H_2_O; PB; pH 7.3), for 24 h at RT. After two washes in PB, samples were post-fixed in 2% osmium tetroxide for 48 h at RT, rinsed in distilled water, and dehydrated in graded ethanol solutions of 50, 70, 80, and 95%, with two final 100% ethanol changes, for 10 min in each dilution. Samples were chemically dried in Hexamethyldisilazane (HMDS) (Sigma-Aldrich Co., St. Louis, MO, United States) by a first incubation in 50% HMDS diluted in absolute ethanol for 24 h, followed by a 15 min incubation in 100% HMDS. Samples were left to air-dry in a fume hood at RT. Dried samples were mounted on an aluminum stub with double-sided adhesive carbon tape and sputter-coated with a thin film of Au/Pd, to improve the electrically conducting properties of the sample surface. Image acquisition was performed using a High resolution (Schottky) Environmental Scanning Electron Microscope with X-Ray Microanalysis and Electron Backscattered Diffraction analysis (FEI Quanta 400 FEG ESEM/EDAX Genesis X4M; Thermo Fisher Scientific).

### Nanoparticle Tracking Analysis of OMVs

The quantification and sizing of the OMVs were determined using a NS300 particle−size tracker with the Nanosight NTA 3.0 software (Malvern, Worcestershire, United Kingdom). Samples were diluted (1:40,000) in saline to achieve a concentration of 10^7^–10^9^ particles/mL for the analysis. Under controlled fluid flow, three measurements (videos of 30 s each) of each sample were acquired as technical replicates and results were averaged. Reads were performed using the camera level adjusted to a value between 14 and 16, and a detection threshold fixed at five. The sample chamber was flushed with sterile PBS between samples, to avoid cross-contamination.

### Protein Quantification of OMVs

A total of 10^11^ OMVs were diluted in 4× Laemmli buffer (Bio-Rad Laboratories Inc.) with β-mercaptoethanol (Sigma-Aldrich), denaturated at 95°C for 5 min and loaded onto 7.5% sodium dodecyl sulfate–polyacrylamide gels (SDS-PAGE). After electrophoresis, gels were stained with 25 mL of BlueSafe (NZYTech, Lisbon, Portugal), for 1 h at RT with gentle rotation, washed with distilled water for 10 min three times, and visualized in a GS-800 Calibrated Densitometer (Bio-Rad Laboratories Inc.). Bands were quantified by densitometry using the Quantity One^®^ 1-D version 4.6.8 software (Bio-Rad Laboratories Inc.). Bovine serum albumin (#05482, Sigma-Aldrich Co.) was used as a protein standard to draw a calibration curve, following the same procedure as OMVs samples. The protein concentration of the OMVs samples was determined by the interpolation from the standard curve. This protocol was optimized by Steeve Lima and Paulo Oliveira at i3S (unpublished).

### Proteomics Analysis

A pellet of 5 × 10^11^ *H. pylori* 26695 OMVs isolated from liquid bacterial cultures at 48, 64, and 72 h of growth was lysed in cold lysis buffer (1% NP-40, 1% Triton X-100 diluted in PBS, pH7.4) containing 1× Bacterial Protease Arrest^TM^ (GBiosciences, St. Louis, MO, United States) and 6 mg/mL lysozyme (PanReac AppliChem S. L. U., Barcelona, Spain), for 1 h on ice. Upon centrifugation (21,000 × *g* for 15 min at 4°C), the cleared lysate was recovered and solubilized in a solution of 100 mM Tris pH 8.5, 1% sodium deoxycholate, 10 mM tris(2-carboxyethyl)phosphine (TCEP), 40 mM chloroacetamide and 1× complete^TM^ protease inhibitor cocktail (Roche Applied Science, Mannheim, Germany), for 10 min, at 95°C, with a constant rotation of 1,000 rpm (Thermomixer, Eppendorf, Hamburg, Germany). Samples were processed for proteomics following the solid-phase-enhanced sample-preparation (SP3) protocol as described in Hughes et al. (2019). Briefly, enzymatic digestion was achieved by adding 2 μg Trypsin/LysC to each sample and incubated overnight at 37°C with constant rotation (1,000 rpm). Protein identification and quantitation was performed by nanoLC-MS/MS using an Ultimate 3000 liquid chromatography system coupled to a Q-Exactive Hybrid Quadrupole-Orbitrap mass spectrometer (Thermo Fisher Scientific). Samples were loaded onto a trapping cartridge (Acclaim PepMap C18 100Å, 5 mm × 300 μm i.d., 160454, Thermo Fisher Scientific) in a mobile phase of 2% acetonitrile (ACN), 0.1% formic acid (FA) at 10 μL/min. After 3 min loading, the trap column was switched in-line to a 50 cm by 75 μm inner diameter EASY-Spray column (ES803, PepMap RSLC, C18, 2 μm, Thermo Fisher Scientific), at 300 nL/min. Separation was generated by gradient mixing A (0.1% FA) and B (80% ACN, 0.1% FA) as follows: 5 min (2.5% B to 10% B), 120 min (10% B to 30% B), 20 min (30% B to 50% B), 5 min (50% B to 99% B), and 10 min (hold 99% B). Subsequently, the column was equilibrated with 2.5% B for 17 min. Data acquisition was controlled by Xcalibur 4.0 and Tune 2.9 software (Thermo Fisher Scientific). The mass spectrometer was operated in data-dependent (dd) positive acquisition mode alternating between a full scan (m/z 380–1,580) and subsequent HCD MS/MS of the 10 most intense peaks from full scan (normalized collision energy of 27%). ESI spray voltage was 1.9 kV. Global settings: use lock masses best (m/z 445.12003), lock mass injection Full MS, chrom. peak width (FWHM) 15s. Full scan settings: 70k resolution (m/z 200), AGC target 3 × 10^6^, maximum injection time 120 ms. dd settings: minimum AGC target 8 × 10^3^, intensity threshold 7.3 × 10^4^, charge exclusion: unassigned, 1, 8, > 8, peptide match preferred, exclude isotopes on, dynamic exclusion 45 s. MS2 settings: microscans 1, resolution 35k (m/z 200), AGC target 2 × 10^5^, maximum injection time 110 ms, isolation window 2.0 m/z, isolation offset 0.0 m/z, spectrum data type profile. Three biological replicates were used for each time point and the LC-MS acquisition of each sample was performed in triplicate.

### Database Searching, Protein Identification, and Classification

Raw data was processed using the Proteome Discoverer 2.5.0.400 software (Thermo Fisher Scientific) and searched against the UniProt^1^ database for the *H. pylori* 26695 Proteome 2021_01 release, 1552 entries, and a common contaminant database from MaxQuant (version 1.6.2.6, Max Planck Institute of Biochemistry, Munich, Germany). The Sequest HT and the MS Amanda 2.0 search engines, together with the Inferys PSM rescoring node, were used to identify tryptic peptides. The ion mass tolerance was 10 ppm for precursor ions and 0.02 Da for fragment ions. Maximum allowed missing cleavage sites was set to 2. Cysteine carbamidomethylation was defined as constant modification. Methionine oxidation, asparagine and glutamine deamidation, peptide N-terminus glutamine to pyro-glutamine, and protein N-terminus acetylation, methionine loss, and methionine loss plus acetylation, were defined as variable modifications. Peptide confidence was set to high. The processing node Percolator was enabled with the following settings: maximum delta Cn 0.05; decoy database search target false discovery rate 1%, validation based on q-value. Protein label free quantitation was performed with the Minora feature detector node at the processing step. Precursor ions quantification was performed at the processing step with the following parameters: unique plus razor peptides were used for quantification, precursor abundance based on intensity and normalization based on total peptide amount. Common contaminants were excluded from data analysis. The ANOVA hypothesis test (individual proteins) for *p*-value calculation was performed for the three bacterial growth periods. Differentially expressed proteins were identified using the following parameters: fold change ratios ± 2.00 and *p* < 0.05. The total abundance of the predicted groups was calculated by the sum of the abundance of proteins classified for each group Prediction of proteins cellular localization and molecular function was obtained from the gene ontology (GO) UniProt database (see text foot note 1). The mass spectrometry proteomics data have been deposited to the ProteomeXchange Consortium via the PRIDE (Perez-Riverol et al., 2019) partner repository with the dataset identifier PXD025393 and 10.6019/PXD025393.

### Statistical Analysis

Data were analyzed using the GraphPad Prism version 8.4.3 software (San Diego, CA, United States). The one-, two-way and Brown-Forsythe ANOVA, with the *post hoc* Tukey’s, Dunnett’s or Sidak’s tests for paired comparisons, were applied for comparisons between three independent groups. Statistically significance was set at *p*-values ≤ 0.05 (*p ≤ 0.05, **p ≤ 0.01, ***p ≤ 0.001, ****p ≤ 0.0001).

## Results

### *Helicobacter pylori* Growth and Viability in F12 Liquid Medium Supplemented With Cholesterol

Considering the goal to isolate OMVs from *H. pylori* cultures grown in a chemically defined medium, we started by characterizing the bacterial growth and viability in the Ham’s F-12 liquid medium supplemented with cholesterol, previously reported to support *H. pylori* growth (Testerman et al., 2001).

**FIGURE 1:**
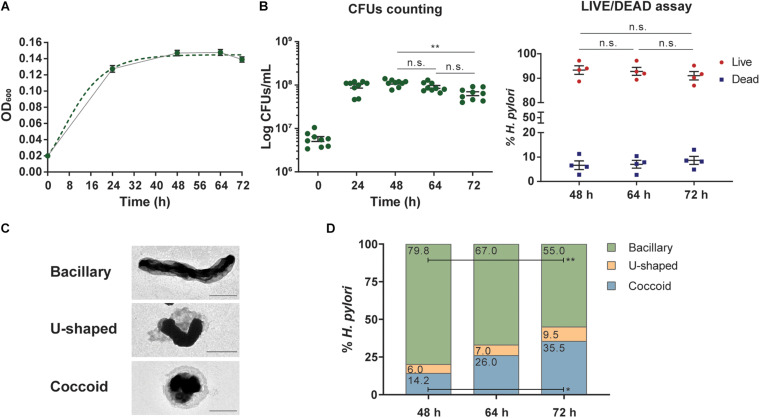
Kinetics, viability, and morphology of *Helicobacter pylori* growth in liquid F-12-cholesterol. **(A)** Growth curve of *Helicobacter pylori* 26695 grown in F12 liquid medium supplemented with 1× cholesterol (continuous line), modeled using the Gompertz growth equation model (dotted line), calculated with GraphPad Prism, and based on the optical density measurements of the bacterial suspension at 600 nm (OD_600_) from 24, 48, 64, and 72 h cultures. Data are shown as mean ± SEM of 16 biological replicates. **(B)** Bacterial viability evaluated by colony-forming units (CFUs) counting (left graph) and the LIVE/DEAD BacLight Bacterial Viability assay (right graph). The number of CFUs was determined at the referred time points, represented as CFUs/mL. Each dot represents a biological replicate (*n* = 9) and data are shown as mean ± SEM. Statistical significance was evaluated using the one-way ANOVA with *post hoc* Tukey’s test, comparing all datasets with each other; only comparisons between 48, 64, and 72 h were illustrated for the sake of simplicity; ^∗∗^*p* ≤ 0.01, n.s. – not significant. LIVE/DEAD BacLight Bacterial Viability assay at 48, 64, and 72 h of growth, by flow cytometry. After sample staining with SYTO9 and PI dies and acquisition on a FACSCanto II cytometer, live and dead bacteria were gated and defined as SYTO9^+^PI^–^ and SYTO9^+^PI^+^, respectively. Each dot represents a biological replicate (*n* = 4) and data are shown as mean ± SEM of the frequency of gated SYTO9^+^PI^–^ and SYTO9^+^PI^+^ bacteria. Statistical significance was evaluated using the two-way ANOVA with *post hoc* Tukey’s test; n.s. – not significant. **(C)** Representative negative stain TEM micrographs of bacillary, U-shaped, and coccoid forms of *H. pylori* from a 64 h liquid culture. Scale bars: 1 μm; 80,000× (bacillary and coccoid) and 8,000× (U-shaped) original magnifications. **(D)** Quantification of bacillary, U-shaped, and coccoid forms of *H. pylori* present in F12-cholesterol liquid cultures at 48, 64, and 72 h from negative stained TEM micrographs, using manual counting. The frequency of each bacterial form was calculated considering the total number of bacteria (741, 1,215, and 1,061) counted in micrographs taken from samples of each time point, 48 h (*n* = 5), 64 h (*n* = 5), and 72 h (*n* = 2), respectively. The mean frequency of each form is displayed inside the respective bar. Statistical significance was evaluated using the two-way ANOVA with *post hoc* Tukey’s test, comparing all datasets with each other; statistical significance was only observed between 72 and 48 h for bacillary and coccoid forms; ^∗^*p* ≤ 0.05 and ^∗∗^*p* ≤ 0.01.

The bacterial growth of *H. pylori* 26695 was monitored by measuring the optical density at 600 nm (OD_600_) of the bacterial liquid cultures at 24, 48, 64, and 72 h, starting with an inoculum density of 0.02 per mL at OD_600_ (∼6 × 10^6^ CFUs/mL) in 200 mL of F12-cholesterol medium (Figure 1A). The growth curve was predicted using the Gompertz model (Mytilinaios et al., 2012) fitted to our data. *H. pylori* presented an exponential growth during the first 32 h of culture, after which it reached a stationary phase that lasted nearly until the endpoint of 72 h. The experimental OD_600_ values at 24 h, 48 h, 64 h, and 72 h were, respectively, 0.127 ± 0.004, 0.147 ± 0.003, 0.148 ± 0.004, and 0.139 ± 0.003, decreasing 6.1% from 64 h to 72 h, although not statistically significant (*p* = 0.3528; one-way ANOVA with *post hoc* Tukey’s test), indicating that bacterial growth might become limited by nutrient availability. The growth kinetics of *H. pylori* 26695 in the chemically defined F12-cholesterol medium and in the complex BB medium supplemented with 5% FBS was compared, under the same experimental conditions (Supplementary Figure 1A). The growth curve was similar for both media, although bacteria grew faster in BB-FBS, with bacterial cultures reaching higher densities (OD_600_ at 24 h: 0.160 ± 0.010; 48 h: 0.199 ± 0.004; 64 h: 0.206 ± 0.011; and 72 h: 0.209 ± 0.005).

To assess the viability of *H. pylori* 26695 strain in F12-cholesterol, we determined the number of CFUs and performed the LIVE/DEAD BacLight Bacterial Viability assay using flow cytometry. The number of CFUs increased until 48 h of bacterial growth (24 h: 9.46 ± 0.96 × 10^7^ CFUs/mL; 48 h: 1.12 ± 0.06 × 10^8^ CFUs/mL), and decreased slightly at 64 h, although not statistically significant (64 h: 9.11 ± 0.71 × 10^7^ CFUs/mL; 48 h *vs.* 64 h: *p* = 0.2080), and until 72 h (6.38 ± 0.66 × 10^7^ CFUs/mL, 48 h *vs.* 72 h: *p* = 0.0082; 64 h *vs.* 72 h: *p* = 0.0571) (Figure 1B). For comparative purposes, we also evaluated the number of CFUs in BB-FBS bacterial cultures (Supplementary Figure 1B). The number of CFUs was similar at 24 h of growth in both media (24 h: 8.47 ± 0.38 × 10^7^ CFUs/mL; *p* = 0.8217), and significantly lower in BB-FBS at 48 h (6.88 ± 0.44 × 10^7^ CFUs/mL; *p* < 0.0001), 64 h (4.58 ± 0.71 × 10^7^ CFUs/mL; *p* < 0.0001), and 72 h (3.00 ± 0.70 × 10^7^ CFUs/mL; *p* = 0.0030). These results show that F12-cholesterol medium sustains the growth and a higher number of viable *H. pylori*, although at a slower rate than BB-FBS medium. The viability of *H. pylori* 26695 grown in F12-cholesterol was also evaluated by another method, the LIVE/DEAD BacLight assay. This assay relies on the simultaneous staining with two fluorescent nucleic acids dyes to distinguish between live and dead bacteria: SYTO9 (green) that enters in all bacterial cells, staining both live and dead cells, and PI (red) that enters in cells with a disrupted membrane, labeling only dead bacteria. Using SYTO9 to define the bacterial cell population, the percentage of live (SYTO9^+^PI^–^) and dead (SYTO9^+^PI^+^) bacteria was then calculated for each sample. No statistically significant differences in the percentage of live (48 h: 93.3 ± 1.8%; 64 h: 92.8 ± 1.6%; 72 h: 91.0 ± 1.7%) and dead (48 h: 6.7 ± 1.8%; 64 h: 7.1 ± 1.6%; 72 h: 8.7 ± 1.7%) bacteria were found between the experimental time points (Figure 1B and Supplementary Figure 2 for gating strategy). This shows that bacteria remained viable until 72 h, despite the above-mentioned decrease in the number of CFUs.

Knowing that *H. pylori* morphology changes from a bacillary to a coccoid structure in response to nutrient deprivation and other adverse environmental conditions (Kusters et al., 1997; Azevedo et al., 2007), which could explain the differences between CFUs counts and LIVE/DEAD assay results, we evaluated the morphological alterations during the bacterial growth in F12-cholesterol. Bacillary, U-shaped, and coccoid bacteria were manually counted from negative stain TEM micrographs taken from 48, 64, and 72 h bacterial liquid cultures (Figure 1C). No statistically significant differences were found regarding the frequency of each morphological shape between samples collected at 48 and 64 h, and between 64 and 72 h samples (Figure 1D). However, a significant decrease in the number of bacillary (*p* = 0.003) and an increase in the number of coccoid *H. pylori* (*p* = 0.012) were observed in samples collected at 72 h when compared to those obtained at 48 h. This observation suggests that coccoid forms are viable but non-culturable (Ierardi et al., 2020), as there was no alteration in the frequency of viable bacteria, assessed by the LIVE/DEAD assay, at this time point. As an ancillary analysis, we checked the morphology of *H. pylori* grown in F12-cholesterol by SEM (Supplementary Figure 3), which confirmed that the bacillary shape was predominant at all time points, with the U-shape and coccoid bacteria becoming noticeable in the 72 h cultures.

Altogether, these results show that the F12-cholesterol medium is capable of nutritionally support *H. pylori* growth, preserving its typical bacillary morphology and viability.

### Quantification and Morphological Characterization of OMVs Secreted by *Helicobacter pylori* Grown in F12-Cholesterol Medium

**FIGURE 2:**
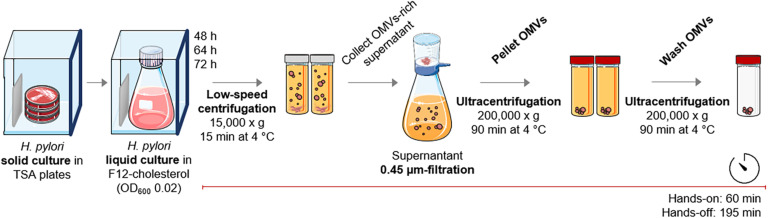
Outline of the OMVs isolation method from *Helicobacterpylori* cultures in F12-cholesterol medium. After the establishment of the liquid bacterial culture, the protocol for OMVs isolation includes one low-speed centrifugation, a 0.45 μm-filtration of the bacteria-free supernatant, and two ultracentrifugations for the recovery and wash of OMVs, comprising 60 min of hands-on period and 195 min of hands-off period (see details in Materials and Methods section). This figure used elements from Servier Medical Art (www.servier.fr/servier-medical-art).

After ensuring that the F12-cholesterol medium supports both growth and viability of *H. pylori* 26695 under our experimental settings, we optimized the OMVs’ isolation protocol to be fast and simple, while reliable. This protocol comprises one low-speed centrifugation followed by a 0.45 μm-filtration to deplete both bacterial cells and debris, one ultracentrifugation to recover OMVs, and a final ultracentrifugation to wash the OMVs fraction (Figure 2). The overall duration of the protocol is of approximately 4 h, distributed in 60 min of hands-on and 195 min of hands-off time. This protocol is substantially shorter than those published, which report the hands-off time between 335 min to 1,170 min (Mullaney et al., 2009; Olofsson et al., 2010).

**FIGURE 3:**
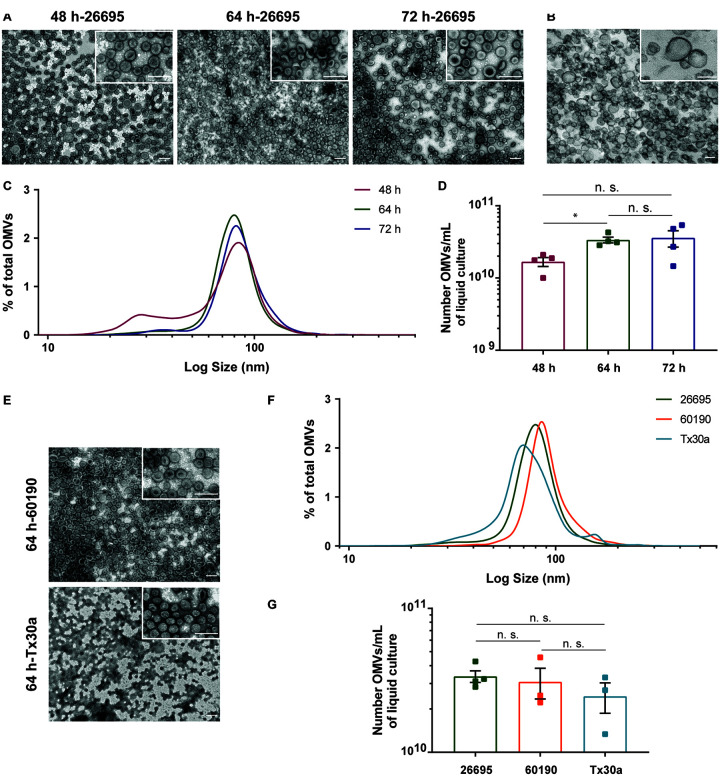
Morphological characterization, size distribution, and yield of OMVs secreted by *Helicobacter pylori* grown in F12-cholesterol medium. **(A)** Negative staining of OMVs isolated from *H. pylori* 26695 F12-cholesterol liquid cultures at 48, 64, and 72 h of growth, and **(B)** ultrastructure section of OMVs from 64 h *H. pylori* cultures. Scale bars: 200 nm; 50,000×, 100,000× [insets in panel **(A)**] and 200,000× [inset in panel **(B)**] original magnifications. **(C)** Size distribution, represented as percentage of the total number of isolated OMVs; data are shown as mean ± SEM of four biological replicates and statistical significance was evaluated using the one-way ANOVA with *post hoc* Tukey’s test; n.s. – not significant. **(D)** Number of recovered OMVs per mL of bacterial culture, determined using Nanoparticle Tracking Analysis (NTA) at 48, 64, and 72 h periods of bacterial growth; data are shown as mean ± SEM of four biological replicates and statistical significance was evaluated using the one-way ANOVA with *post hoc* Tukey’s test; ^∗^*p* ≤ 0.05, n.s. – not significant. **(E)** Representative negative stain TEM micrographs of *H. pylori* 60190 and Tx30a-OMVs generated from 64 h F12-cholesterol bacterial cultures. Scale bars: 200 nm; 50,000× and 100,000× (insets) original magnification. **(F)** Size distribution, represented as percentage of the total number of isolated OMVs at 64 h of liquid culture, and **(G)** number of recovered OMVs per mL of bacterial culture determined by NTA; data are shown as mean ± SEM of 4 (for 26695) or 3 (for 60190 and Tx30a) biological replicates and statistical significance was evaluated using the Brown-Forsythe and Welch ANOVA with *post hoc* Dunnett’s test; n.s. – not significant.

The OMVs recovered following this protocol exhibited a spherical shape with a central depression and a heterogeneous size, as visualized by negative staining followed by TEM (Figure 3A), matching the OMVs’ prototypical morphology and size distribution (Chutkan et al., 2013). Moreover, ultrastructural analysis enabled validation of the vesicles as OMVs, since they are delimited by a single lipid bilayer (Figure 3B). TEM analysis revealed the absence of bacterial debris, flagella, and proteins, showing that OMVs preparations were highly pure. The comparative analysis of the negative staining images of OMVs isolated from *H. pylori* 26695 grown in F12-cholesterol and in complex BB-FBS medium, and between the respective culture media alone, highlighted the importance of using the synthetic medium for the recovery of highly pure OMVs. F12-cholesterol medium and OMVs isolated from F12-cholesterol bacterial cultures had a clear background, without protein aggregates (Figure 3A and Supplementary Figure 4A), distinctly from BB-FBS medium and the respective OMVs samples, in which the presence of proteins was detected (Supplementary Figures 4B,C).

We next characterized the size distribution and number of OMVs recovered from the 48, 64, and 72 h *H. pylori* cultures by NTA (Figure 3C). More than 99% of all OMVs presented a size range between 20 and 200 nm, regardless of the culture time, and 0.66% of vesicles were detected above the 200 nm. The presence of OMVs higher than 450 nm was negligible, which matched the exclusion filter pore-size and demonstrated the absence of vesicle aggregates. From the NTA data, we also identified that the mode sizes of OMVs isolated from the 48, 64, and 72 h liquid cultures were, respectively, 84.3 ± 1.7, 79.8 ± 6.4, and 83.3 ± 5.0 nm. Despite the fact that OMVs isolated from 48 h bacterial cultures were more heterogeneous in size than OMVs from 64 and 72 h cultures, presenting a distinctive population of smaller OMVs with a diameter ranging from 15 to 60 nm, the size distribution of OMVs from the three time points presented no statistically significant differences between them.

Concerning the yield of OMVs, an average of 1.68 ± 0.24 × 10^10^, 3.37 ± 0.31 × 10^10^, and 3.59 ± 0.91 × 10^10^ OMVs per mL of liquid culture was recovered from the 48, 64, and 72 h bacterial cultures, respectively (Figure 3D). The 64 h bacterial cultures produced a significantly higher number of OMVs/mL when compared to the 48 h cultures (*p* = 0.014), but not significantly different from the 72 h cultures. Although we cannot exclude some loss of OMVs during the isolation procedure, we assume that the number of secreted vesicles is likely near the recovered ones, given that no vesicles were detected in the supernatant collected after the first ultracentrifugation, both in F12-cholesterol and in BB-FBS, when analyzed by TEM (Supplementary Figures 4D,E). As so, the shorter number of steps and the recovery of a high yield of vesicles emphasize the efficacy of our protocol, even when different culture media are used.

To ascertain the applicability of this protocol to *H. pylori* strains other than 26695, we selected two additional *H. pylori* reference strains, 60190 and Tx30a. These strains were grown in F12-cholesterol medium, under the same conditions as strain 26695, and their OMVs were isolated and characterized by TEM and NTA, in the 64 h bacterial cultures. OMVs recovered from *H. pylori* 60190 and TX30a were highly pure and with the same size heterogeneity as 26695-OMVs (Figures 3E,F). The number of OMVs recovered per mL of bacterial culture was similar between the three strains at 64 h of bacterial growth (26695: 3.37 ± 6.27 × 10^9^; 60190: 3.09 ± 1.29 × 10^10^; Tx30a: 2.45 ± 1.01 × 10^10^ OMVs/mL) (Figure 3G).

In summary, these results show that OMVs recovered from *H. pylori* grown in F12-cholesterol cultures are *bona fide*, presenting the same morphology and ultrastructure as those isolated from complex media (Chutkan et al., 2013; Turner et al., 2018). Furthermore, our abridged protocol provided a good yield and a highly pure population of OMVs from various *H. pylori* strains and in different phases of bacterial growth.

### Proteomic Content and Proteomic Analysis of OMVs Isolated From *Helicobacter pylori* 26695 Grown in F12-Cholesterol Culture Medium

**FIGURE 4:**
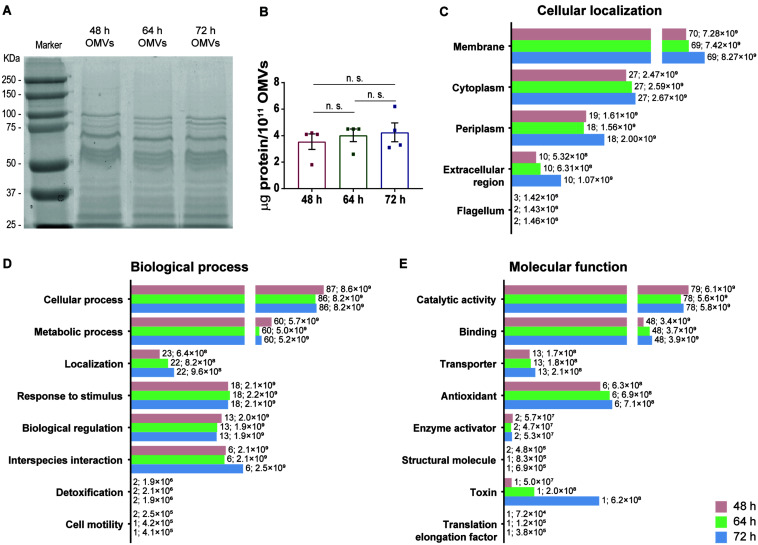
Protein profile and proteomic analysis of OMVs secreted by *Helicobacter pylori* grown in F12-cholesterol medium. **(A)** Protein profile of 10^11^ OMVs isolated from 48, 64, and 72 h-*H. pylori* 26695 bacterial cultures, after staining a SDS-PAGE with BlueSafe, and corresponding **(B)** protein quantification. Data are shown as mean ± SEM of four biological replicates and statistical significance was evaluated using the one-way ANOVA with *post hoc* Tukey’s test; n.s. – not significant. Proteomic analysis of 48, 64, and 72 h-OMVs isolated from *H. pylori* 26695 F12-cholesterol liquid cultures by nanoLC-MS/MS and prediction of the **(C)** cellular localization, **(D)** biological process, and **(E)** molecular function of the identified proteins using the gene ontology UniProt database. Data are shown as the abundance of proteins in each group; for each bar, the number of proteins and respective abundance is indicated.

The next aim was to characterize and compare the protein cargo of OMVs secreted by *H. pylori* grown in F12-cholesterol at different time points of bacterial growth. The total protein amount from OMVs was assessed by SDS-PAGE and gel staining with BlueSafe, using lysates from 10^11^ OMVs (Figure 4). OMVs isolated from 48, 64 and 72 h bacterial cultures presented an identical protein profile (Figure 4A) and similar total protein amounts (48 h: 3.55 ± 1.16; 64 h: 4.02 ± 0.95; 72 h: 4.25 ± 1.42 μg of protein per 10^11^ OMVs) (Figure 4B).

Next, we applied nanoscale liquid chromatography coupled to tandem mass spectrometry (nanoLC-MS/MS) to 5 × 10^11^ OMVs of each time point of bacterial growth, and analyzed the differential expression of proteins between each of them. A total of 267, 268 and 269 proteins from *H. pylori* 26695 were identified on 48, 64, and 72 h-OMVs, respectively (Supplementary Table 1). Of these, 233, 231, and 232 proteins with a false discovery rate of 1% were further classified according to their predicted cellular localization, biological process and molecular function, using the GO UniProt database resource and manual curation (Supplementary Table 2). All proteins identified in 64 h-OMVs (231) were common to 48 and 72 h samples. Pyruvate ferredoxin oxidoreductase was identified only in 48 and 72 h samples, whereas flagellar P-ring protein was detected exclusively in 48 h-OMVs.

In terms of cellular localization (Figure 4C), the most diverse and abundant group of proteins identified in OMVs from all time points analyzed was predicted to be associated with the bacterial membrane, and within this group outer membrane proteins (OMPs) were the most frequent (48 h: *n* = 50, abundance = 5.67 ± 0.41 × 10^9^; 64 h: *n* = 49, abundance = 5.73 ± 1.25 × 10^9^; 72 h: *n* = 49, abundance = 6.52 ± 0.74 × 10^9^). Periplasmic, cytoplasmic and extracellular region-associated proteins were also detected. In addition, 3 flagellar proteins were identified in 48 h samples, while only 2 were detected in 64 and 72 h-OMVs. Considering that OMVs originate from the blebbing of the OM, a high abundance of membrane-associated proteins was expected, and is in agreement with previous proteomic data from *H. pylori* 26695 OMVs (Zavan et al., 2019).

Concerning the biological process analysis, proteins were distributed into eight groups (Figure 4D). Proteins involved in cellular processes constituted the most numerous and abundant group, followed by proteins involved in metabolic processes, localization proteins, response to stimulus, biological regulation, and interspecies interaction proteins. Detoxification and cell motility proteins were the less abundant.

Regarding the molecular functions (Figure 4E), the majority of the classified proteins was predicted to have catalytic, binding and/or transporter activities, followed by proteins with antioxidant and structural molecular activities, enzyme activators, a toxin, and a translation elongation factor. Besides the above-mentioned molecular functions, several of the identified proteins are also involved in processes related with *H. pylori* colonization, survival, and pathogenesis. In particular, urease α/β neutralizes the acidic environment of the gastric mucosa (Marshall et al., 1990), which together with outer membrane proteins (BabA and OipA), contributes to *H. pylori* colonization (Ishijima et al., 2011; Teymournejad et al., 2017); β-lactamase (HcpA, HcpC, HcpD, and HcpE) provides resistance to amoxicillin (Tseng et al., 2009); HtrA disrupts the epithelial cell-cell junctions, enabling transmigration of *H. pylori* across the gastric epithelium (Tegtmeyer et al., 2017); VacA and GGT induce, vacuolization and apoptosis (Ricci et al., 1997; Shibayama et al., 2003); HP-NAP, OipA, and urease β act as immune modulators by inducing pro-inflammatory responses (Yamaoka et al., 2002; de Bernard and D’Elios, 2010; Schoep et al., 2010); catalase, superoxide dismutase and thioredoxin protect *H. pylori* from the oxidative stress (Pesci and Pickett, 1994; Odenbreit et al., 1996; Kuhns et al., 2015); HP-NAP and bacterial non-heme ferritin are iron storage proteins, a key element for bacterial survival (Ge and Sun, 2012). The presence of these virulence factors in *H. pylori* OMVs isolated from F12-cholesterol and from other complex liquid cultures (Mullaney et al., 2009; Olofsson et al., 2010; Zavan et al., 2019) denotes that some proteins may be preferably loaded onto OMVs, independently of the culture media or bacterial strain, and highlights the importance of OMVs for the success of *H. pylori* infection. Seventy of the 233 identified proteins were uncharacterized and approximately half of them did not have a predicted cellular localization or molecular function.

Although OMVs isolated from 48, 64, and 72 h-bacterial cultures had a similar protein diversity, some of the proteins were differentially expressed (Supplementary Table 3 for the comparison between 48 and 64 h; Supplementary Table 4 for the comparison between 48 and 72 h; Supplementary Table 5 for the comparison between 64 and 72 h). In particular, the virulence factor VacA was found to be significantly more abundant in 64 and 72 h-OMVs, differently from the data of Zavan et al. (2019) that shows an enrichment of this protein in 16 h-OMVs in comparison to vesicles isolated from 48 h and 72 h bacterial cultures. Additionally, the thioredoxin reductase, which protects *H. pylori* from the oxidative stress (Kuhns et al., 2015), and 2 proteins involved in iron acquisition and transport across the membrane, which is an essential micronutrient for bacterial survival, FrpB (Gonzalez-Lopez and Olivares-Trejo, 2009) and FecA, presented a significantly higher expression in 64 and 72 h-OMVs, in comparison to 48 h samples.

Overall, our proteomic analysis confirms the reliability of the protocol, by showing that the cargo of OMVs contain proteins from the membrane, periplasmic, and cytoplasmic bacterial compartments, and supports the selective sorting of protein cargo into OMVs, as only a fraction of the total bacterial proteins is represented in the cargo of OMVs.

## Discussion

Outer membrane vesicles have emerged as an important delivery system of bacterial components with an impact on bacteria-bacteria and bacteria-host interactions. In addition, OMVs are increasingly used for diverse therapeutic applications, namely vaccine development, taking advantage of their flexibility to genetic manipulation and nanosized structure for dissemination throughout the body, besides their easy production at large-scale and reduced costs (Bitto and Kaparakis-Liaskos, 2017).

The prevailing methods for the isolation of *H. pylori* OMVs are time-consuming and diverse, hampering the standardization of a suitable protocol for OMVs isolation, which is essential for downstream research and biomedical applications, namely the assessment of the functional roles of OMVs *in vitro* and *in vivo*, and large-scale production. The current published protocols have various disadvantages. First, they rely on the use of complex undefined media that contain yeast and animal tissue extracts, thus affecting the purity, content, and variability of OMVs between samples (Olofsson et al., 2010; Tsolakos et al., 2010). Second, they have a lengthy duration due to the multiple centrifugation steps, which might compromise the final yield of OMVs (Chutkan et al., 2013). Finally, the high variability of procedures and diversity of protocols weakens the comparison between studies, as the recovered fractions of OMVs can be different.

To overcome these drawbacks, we developed an isolation method of *H. pylori* OMVs trimming non-essential steps after selecting F12 supplemented with cholesterol, a chemically defined medium, as the bacterial culture medium for *H. pylori* growth. The final layout of this method included one low-speed centrifugation followed by supernatant 0.45 μm-filtration to remove bacteria and debris from the culture medium and to collect all range of OMVs sizes, and finally two ultracentrifugations for the recovery and washing of the OMVs.

Our results showed that the F12-cholesterol medium was effective in sustaining bacterial growth, while achieving higher viability than the complex BB-FBS medium. Like previously described for other bacterial culture media (Kusters et al., 1997; Adams et al., 2003; Olofsson et al., 2010), we observed a morphological change of *H. pylori* from the bacillary to the coccoid forms throughout bacterial growth. The significant decrease in the number of CFUs at 72 h was not mirrored by the loss of bacterial viability using the LIVE/DEAD assay, likely due to the fact that coccoid forms are viable but non-culturable bacteria, with minimal metabolic activity, and preserved membrane and genetic material integrities (Adams et al., 2003; Ierardi et al., 2020).

Associated with the bacterial growth was the increased number of secreted OMVs, in accordance with descriptions of OMVs isolated from complex liquid cultures (Olofsson et al., 2010; Zavan et al., 2019). Over 99% of OMVs measured between 20 and 200 nm, with mode of nearly 80 nm, which is distinct from the enrichment in 100 to 200 nm sized OMVs isolated from *H. pylori* grown in BHI supplemented with 0.2% β-cyclodextrin (Zavan et al., 2019). This difference might be related with the distinct medium used, as well as with the effect of β-cyclodextrin, which chelates cholesterol that is essential for *H. pylori* growth (Hutton et al., 2010; Jimenez-Soto et al., 2012), highlighting how bacterial growth conditions impact on the properties of OMVs. Our findings also demonstrated the reproducibility of this protocol, as OMVs isolated from different *H. pylori* strains presented a similar size heterogeneity and were equally pure.

OMVs isolated from F12-cholesterol medium using our shorter protocol were highly pure, free from protein aggregates, flagella, and other bacterial contaminants, while requiring significantly less hands-on/hands-off time. Of relevance was also the high yield of OMVs, with around 10^10^ OMVs per mL of bacterial culture. Moreover, the proteomic analysis of our OMVs confirmed that their cargo contained proteins from several bacterial compartments (e.g., membrane, periplasm, and cytoplasm) and with diverse biological functions, in particular known bacterial virulence factors, already described for different *H. pylori* strains (Jarzab et al., 2020), reinforcing their reliability. Besides the common proteins identified between our and other OMVs’ proteomes (Mullaney et al., 2009; Olofsson et al., 2010; Zavan et al., 2019), there were also unique proteins ascribed to each one. Such differences in OMVs protein cargo might be related to the underlying growth conditions (bacterial strain, culture media, and initial bacterial density), which influence bacterial growth and the vesiculation process, in addition to technical issues (isolation method and proteomic analysis).

Considering the therapeutic potential of bacterial OMVs, it is of uttermost importance that the harvest of OMVs occurs from a chemically defined bacterial culture without animal-derived components that hinder the purification process and ultimately interfere with the host immune response (van der Pol et al., 2015). Our method overcomes this issue and, by having increased time-efficiency in comparison to previous protocols, is a suitable approach for application in the biomedical field. Still, the yield and purity of *H. pylori* OMVs on a large-scale implementation of the protocol, and its application for the isolation of OMVs from other bacteria species, needs further evaluation. Currently, OMVs have been licensed for human use as adjuvant (Nokleby et al., 2007) or vaccine against the serogroup B *Neisseria meningitides*, namely the Bexsero^®^ (Novartis) and VA-MENGOC-BC^®^ (Finlay Institute, Cuba) vaccines (Petousis-Harris, 2018), and other OMVs-based vaccines against infectious diseases are already in preclinical studies (Zhang et al., 2019). In the case of *H. pylori*, the development of an effective prophylactic or therapeutic vaccine, predominantly composed of purified or recombinant components of *H. pylori* antigens with an adjuvant, has proven challenging and not yet a reality (Sutton and Boag, 2019). Promising data showed that intragastric immunization with OMVs from *H. pylori* 7.13, resulted in the development of a specific systemic immune response in mice, and in the significant reduction of bacterial load after challenging with *H. pylori* SS1 (Liu et al., 2019; Song et al., 2020).

In conclusion, we described an abridged protocol for isolation of OMVs resorting to a synthetic chemically defined liquid medium for bacterial growth ensuing a high yield of pure and *bona fide H. pylori* OMVs, in a time- and cost-efficient manner, suitable for research, biomedical, and biopharmaceutical downstream applications.

## Data Availability Statement

The mass spectrometry proteomic data presented in the study is deposited in the ProteomeXchange Consortium via the PRIDE partner repository with the dataset identifier PXD025393 and doi: 10.6019/PXD025393.

## Author Contributions

JM and ML conceptualized the study. JM, VP, TF, AM, and HO acquired the data. JM, VP, TF, AM, HO, CF, and ML performed the data analysis and interpretation. JM drafted the manuscript. All authors revised the manuscript for important intellectual content. CF acquired the funding.

## Conflict of Interest

The authors declare that the research was conducted in the absence of any commercial or financial relationships that could be construed as a potential conflict of interest.

## Footnotes

1 www.uniprot.org
