# Supplementary material for: Isolation Method and Characterization of Outer Membranes Vesicles of Helicobacter pylori Grown in a Chemically Defined Medium
Source: Front Microbiol. 2021 Jun 2;12:654193. doi: 10.3389/fmicb.2021.654193 (PMC8206784; doi:10.3389/fmicb.2021.654193)
Supplement: Supplementary file 1 [file Data_Sheet_1.docx]

Supplementary Material

Isolation method and characterization of outer membranes vesicles of *Helicobacter pylori* grown in a chemically defined medium

**This Supplementary Material File contains:**

Supplementary Figures 1 to 4

Supplementary Tables 3 to 5

For Supplementary Tables 1 and 2, please see Excel File.

# Supplementary Figures


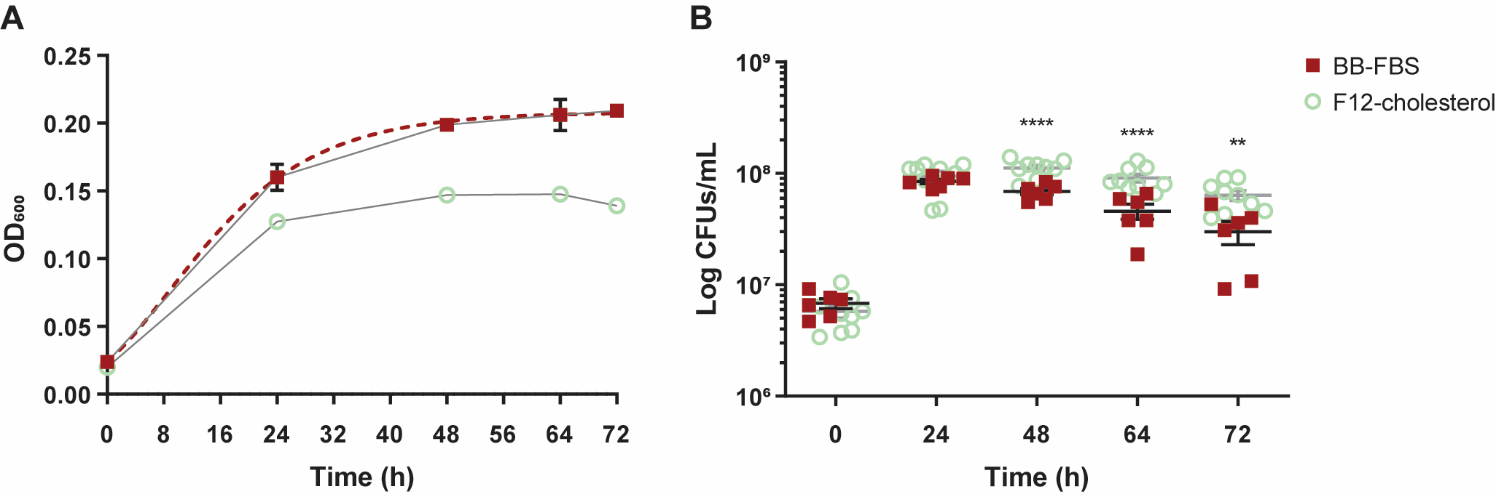
**Supplementary Figure 1. (A)** Growth curve of *H. pylori* 26695 grown in BB liquid medium supplemented with 5x FBS (continuous line defined by the red squares), modeled using the Gompertz growth equation model (red dotted line), calculated with GraphPad Prism, based on the optical density measurements of the bacterial suspension at 600 nm (OD_600_) at 24 h, 48 h, 64 h, and 72 h of culture. Data are shown as mean ± SEM of 6 biological replicates. The growth curve of *H. pylori* 26695 grown in F12-cholesterol is represented for comparison and is the same as in Figure 1A. **(B)** Viable bacteria were determined at the referred time points by CFUs counting and are represented as CFUs/mL. Each dot represents a biological replicate (n=6) and data are shown as mean ± SEM. Statistical significance was evaluated using the two-way ANOVA with post-hoc Sidak’s test. ** *p* ≤ 0.01; **** *p* ≤ 0.0001. The number of CFUs/mL in F12-cholesterol is represented for comparison and is the same as in Figure 1B.

**
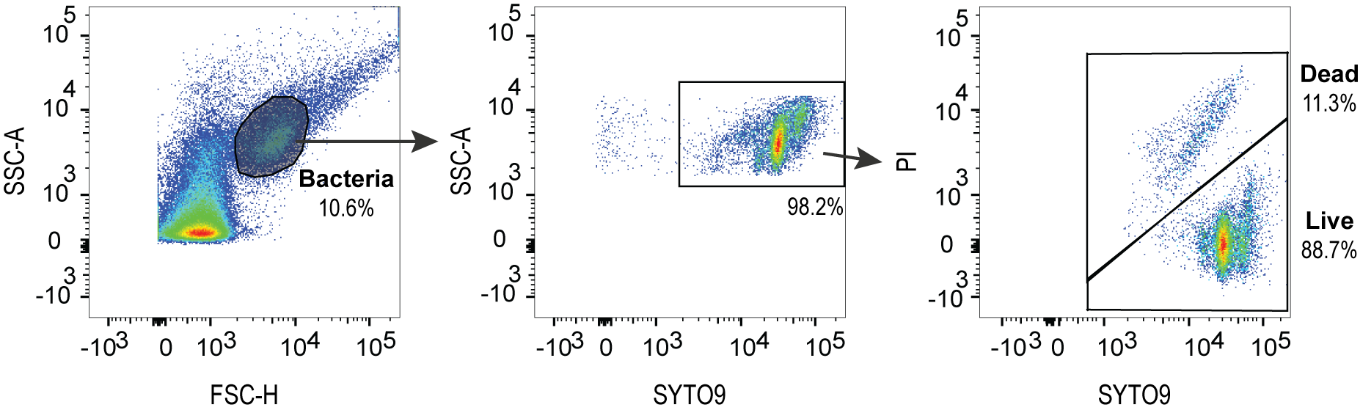
**

**Supplementary Figure 2.** Flow cytometry gating strategy used to define and quantify live and dead *H. pylori* grown for 48 h, 64 h, and 72 h in F12-cholesterol liquid medium. Samples were stained using SYTO9 and PI dyes, acquired in a FACS Canto II cytometer, and analyzed using the FlowJo™ software. Only stained bacterial cells (SYTO9^+^) were considered and two populations of live (SYTO9^+^PI^-^) and dead (SYTO9^+^PI^+^) cells were defined.

**
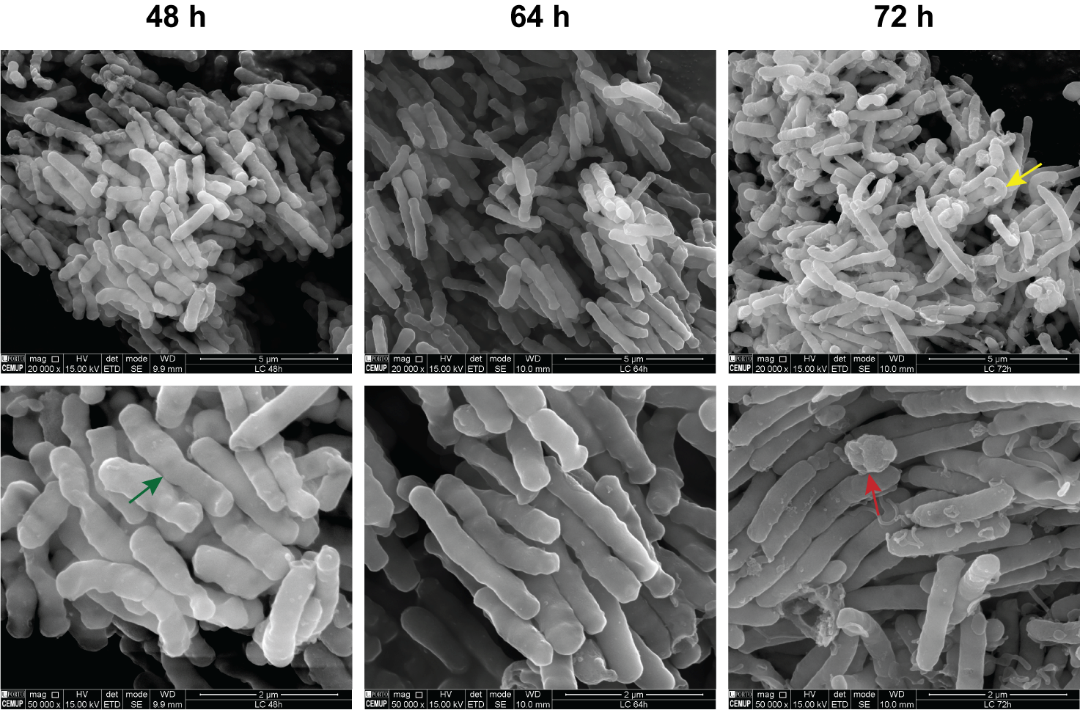
**

**Supplementary Figure 3.** Representative scanning electron micrographs of 48 h, 64 h, and 72 h *H. pylori* liquid cultures. Bacillary (green arrow), U-shapped (yellow arrow) and coccoid (red arrow) forms of *H. pylori* are represented. Top images: scale bars = 5 µm; 20,000x original magnification. Bottom images: scale bars = 2 µm; 50,000x original magnification.

**
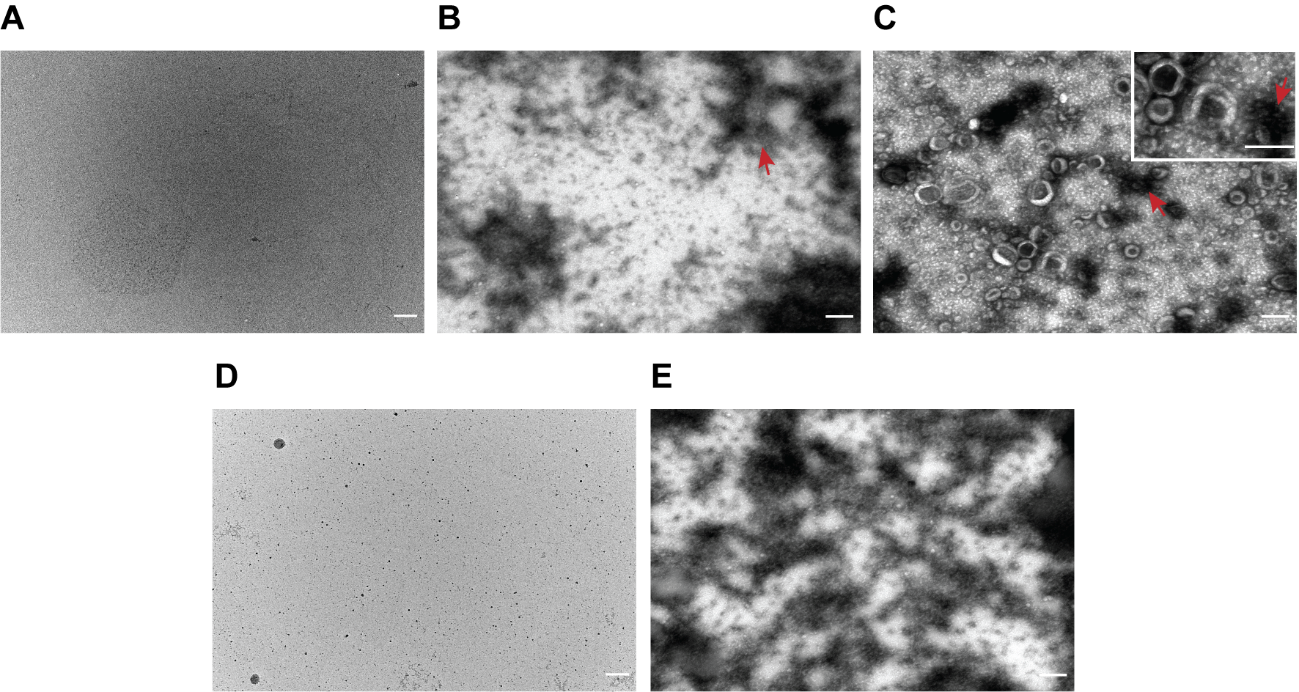
**

**Supplementary Figure 4.** Representative negative stain TEM micrographs of **(A)** F12-cholesterol liquid medium, **(B)** BB liquid medium supplemented with 5% FBS (BB-5% FBS), **(C)** OMVs isolated from BB-5% FBS liquid cultures and the supernatants collected after the first ultracentrifugation from **(D)** F12-cholesterol and **(E)** BB-5% FBS *H. pylori* 26695 liquid cultures. Red arrows point to proteins present in BB-5% FBS liquid medium and in OMVs preparations from BB-5% FBS liquid cultures. Scale bars: 200 nm; **(A**, **B**, **C**, **D** and **E)** 50,000x original magnification and 100,000x (inset) original magnifications.

# Supplementary Tables

**Supplementary Table 3.** Differentially expressed proteins identified in OMVs isolated from 48 h and 64 h *H. pylori* F12-cholesterol liquid cultures.

| **Accession number** | **Protein names** | **Abundance Ratio**  **64 h/48 h** | ***p*-value**  **64 h/48 h** |
| --- | --- | --- | --- |
| P56463 | Purine nucleoside phosphorylase DeoD-type (PNP) (EC 2.4.2.1) | 12.269 | 3.96×10^-5^ |
| O25325 | Uroporphyrinogen decarboxylase (UPD) (URO-D) (EC 4.1.1.37) | 4.794 | 0.001 |
| O25573 | Iron-regulated outer membrane protein (FrpB) | 4.486 | 0.012 |
| P55981 | Vacuolating cytotoxin autotransporter [Cleaved into: Vacuolating cytotoxin; Vacuolating cytotoxin translocator] | 3.946 | 0.012 |
| O25218 | Outer membrane protein (Omp11) | 2.777 | 0.003 |
| P48285 | Enolase (EC 4.2.1.11) (2-phospho-D-glycerate hydro-lyase) (2-phosphoglycerate dehydratase) | 2.454 | 0.013 |
| O25436 | Uncharacterized aminotransferase HP_0736 (EC 2.6.1.-) | 2.129 | 0.046 |
| O25401 | Uncharacterized protein | 2.098 | 0.031 |
| O25395 | Iron(III) dicitrate transport protein (FecA) | 2.074 | 0.014 |
| O25972 | Ribosomal RNA small subunit methyltransferase A (EC 2.1.1.182) (16S rRNA (adenine(1518)-N(6)/adenine(1519)-N(6))-dimethyltransferase) (16S rRNA dimethyladenosine transferase) (16S rRNA dimethylase) (S-adenosylmethionine-6-N'. N'-adenosyl(rRNA) dimethyltransferase) | 2.004 | 0.047 |
| O25410 | Outer membrane protein (Omp15) | 1.927 | 0.031 |
| P56431 | Thioredoxin reductase (TRXR) (EC 1.8.1.9) | 1.920 | 0.038 |
| O25509 | Uncharacterized protein | 1.852 | 0.013 |
| P56089 | Serine hydroxymethyltransferase (SHMT) (Serine methylase) (EC 2.1.2.1) | 1.821 | 0.040 |
| O25884 | YtkA domain-containing protein | 1.784 | 0.032 |
| O25713 | Uncharacterized protein | 1.529 | 0.019 |
| O24863 | Uncharacterized protein | 1.343 | 0.016 |
| O25423 | Uncharacterized protein | 0.918 | 0.050 |
| O25717 | Uncharacterized protein | 0.892 | 0.031 |
| O25270 | Cag pathogenicity island protein (Cag16) | 0.850 | 0.006 |
| O25728 | Putative beta-lactamase HcpC (EC 3.5.2.6) (Cysteine-rich protein C) | 0.841 | 0.048 |
| O24996 | Uncharacterized protein | 0.812 | 0.001 |
| O25872 | Conserved hypothetical secreted protein | 0.796 | 0.046 |
| O25873 | Conserved hypothetical secreted protein | 0.784 | 0.001 |
| P55969 | Neuraminyllactose-binding hemagglutinin (Flagellar sheath adhesin) (N-acetylneuraminyllactose-binding fibrillar hemagglutinin receptor-binding subunit) (NLBH) | 0.782 | 0.024 |
| O24943 | Uncharacterized protein | 0.778 | 0.042 |
| O25271 | Cag pathogenicity island protein (Cag17) | 0.771 | 0.005 |
| O25038 | Conserved hypothetical secreted protein | 0.754 | 0.028 |
| O25137 | Uncharacterized protein | 0.736 | 0.001 |
| O25696 | Uncharacterized protein | 0.718 | 0.017 |
| P56112 | Putative peptidyl-prolyl cis-trans isomerase HP_0175 (PPIase HP_0175) (EC 5.2.1.8) (Rotamase HP_0175) | 0.683 | 0.042 |
| O24938 | Uncharacterized protein | 0.623 | 0.001 |
| O25403 | N-methylhydantoinase | 0.069 | 0.019 |
| O25402 | Hydantoin utilization protein A (HyuA) | 0.049 | 0.016 |
| O25404 | Uncharacterized protein | 0.025 | 0.025 |

**Supplementary Table 4.** Differentially expressed proteins identified in OMVs isolated from 48 h and 72 h *H. pylori* F12-cholesterol liquid cultures.

| **Accession number** | **Protein names** | **Abundance Ratio**  **72 h/48 h** | ***p*-value**  **72 h/48 h** |
| --- | --- | --- | --- |
| P56463 | Purine nucleoside phosphorylase DeoD-type (PNP) (EC 2.4.2.1) | 16.491 | 1.59×10^-5^ |
| P55981 | Vacuolating cytotoxin autotransporter [Cleaved into: Vacuolating cytotoxin; Vacuolating cytotoxin translocator] | 12.376 | 0.002 |
| O25325 | Uroporphyrinogen decarboxylase (UPD) (URO-D) (EC 4.1.1.37) | 6.148 | 2.83×10^-4^ |
| O25573 | Iron-regulated outer membrane protein (FrpB) | 5.620 | 0.015 |
| P56003 | Elongation factor Tu (EF-Tu) | 5.317 | 0.030 |
| O25349 | Quinone-reactive Ni/Fe hydrogenase. large subunit (HydB) | 3.725 | 0.018 |
| O25218 | Outer membrane protein (Omp11) | 3.113 | 0.003 |
| O25329 | Uncharacterized protein | 3.064 | 0.032 |
| Q48252 | CAG pathogenicity island protein 23 (Protein PicB) = CagE | 2.926 | 0.042 |
| O25477 | Uncharacterized protein | 2.727 | 0.048 |
| O26003 | Uncharacterized protein | 2.666 | 0.022 |
| O25230 | Uncharacterized protein | 2.629 | 0.034 |
| O25395 | Iron(III) dicitrate transport protein (FecA) | 2.492 | 0.005 |
| O25410 | Outer membrane protein (Omp15) | 2.327 | 0.008 |
| O25401 | Uncharacterized protein | 2.100 | 0.008 |
| O25713 | Uncharacterized protein | 1.997 | 0.006 |
| P48285 | Enolase (EC 4.2.1.11) (2-phospho-D-glycerate hydro-lyase) (2-phosphoglycerate dehydratase) | 1.989 | 0.019 |
| O25735 | Outer membrane protein (Omp23) | 1.915 | 0.007 |
| P56089 | Serine hydroxymethyltransferase (SHMT) (Serine methylase) (EC 2.1.2.1) | 1.785 | 0.031 |
| P50610 | Flagellar hook protein FlgE | 1.662 | 0.049 |
| O25509 | Uncharacterized protein | 1.628 | 0.018 |
| P56431 | Thioredoxin reductase (TRXR) (EC 1.8.1.9) | 1.617 | 0.035 |
| O25580 | Outer membrane protein (Omp22) | 1.528 | 0.030 |
| O24863 | Uncharacterized protein | 1.329 | 0.043 |
| O25075 | Alginate_lyase domain-containing protein | 1.314 | 0.034 |
| O25442 | Uncharacterized protein | 1.219 | 0.040 |
| O25270 | Cag pathogenicity island protein (Cag16) = CagM | 0.819 | 0.002 |
| P55969 | Neuraminyllactose-binding hemagglutinin (Flagellar sheath adhesin) (N-acetylneuraminyllactose-binding fibrillar hemagglutinin receptor-binding subunit) (NLBH) | 0.805 | 0.017 |
| O25728 | Putative beta-lactamase HcpC (EC 3.5.2.6) (Cysteine-rich protein C) | 0.800 | 0.010 |
| O25076 | YceI domain-containing protein | 0.787 | 0.030 |
| O24943 | Uncharacterized protein | 0.772 | 0.043 |
| O25140 | Thiol:disulfide interchange protein (DsbC). putative | 0.767 | 0.037 |
| O25872 | Conserved hypothetical secreted protein | 0.742 | 0.011 |
| O25271 | Cag pathogenicity island protein (Cag17) = CagN | 0.728 | 0.002 |
| O25696 | Uncharacterized protein | 0.718 | 0.019 |
| O25905 | Protease | 0.707 | 0.025 |
| O25873 | Conserved hypothetical secreted protein | 0.697 | 8.98×10^-5^ |
| P56112 | Putative peptidyl-prolyl cis-trans isomerase HP_0175 (PPIase HP_0175) (EC 5.2.1.8) (Rotamase HP_0175) | 0.675 | 0.019 |
| O24996 | Uncharacterized protein | 0.663 | 7.69×10^-6^ |
| O25018 | UPF0323 lipoprotein HP_0232 | 0.638 | 0.031 |
| O25052 | ATP-dependent nuclease (AddB) | 0.629 | 0.018 |
| O24938 | Uncharacterized protein | 0.577 | 4.35×10^-4^ |
| O25256 | Beta-lactamase (EC 3.5.2.6) | 0.417 | 0.004 |
| O25403 | N-methylhydantoinase | 0.085 | 0.018 |
| O25402 | Hydantoin utilization protein A (HyuA) | 0.068 | 0.021 |
| O25404 | Uncharacterized protein | 0.038 | 0.022 |

**Supplementary Table 5.** Differentially expressed proteins identified in OMVs isolated from 64 h and 72 h *H. pylori* F12-cholesterol liquid cultures.

| **Accession number** | **Protein names** | **Abundance Ratio**  **72 h/64 h** | ***p*-value**  **72 h/64 h** |
| --- | --- | --- | --- |
| O26055 | Uncharacterized protein | 1.408 | 0.015 |
| O25137 | Uncharacterized protein | 1.205 | 0.024 |
| O25495 | Uncharacterized protein | 1.137 | 0.036 |
| O25945 | Outer membrane protein (Omp30) | 1.118 | 0.050 |
| O25423 | Uncharacterized protein | 1.106 | 0.023 |
| O25873 | Conserved hypothetical secreted protein | 0.889 | 0.020 |
| O24996 | Uncharacterized protein | 0.816 | 3.08×10^-4^ |
| O25256 | Beta-lactamase (EC 3.5.2.6) | 0.511 | 0.009 |
